# Plasma concentrations of osteopontin, but not thrombin-cleaved osteopontin, are associated with the presence and severity of nephropathy and coronary artery disease in patients with type 2 diabetes mellitus

**DOI:** 10.1186/1475-2840-9-70

**Published:** 2010-10-29

**Authors:** Xiaoxiang Yan, Motoaki Sano, Lin Lu, Wei Wang, Qi Zhang, Ruiyan Zhang, Lingjie Wang, Qiujing Chen, Keiichi Fukuda, Weifeng Shen

**Affiliations:** 1 Department of Cardiology, Rui Jin Hospital, Jiaotong University School of Medicine, Shanghai 200025, China; 2 Institute of Cardiovascular Diseases, Jiaotong University School of Medicine, Shanghai 200025, China; 3 Division of Cardiology, Department of Medicine, Keio University School of Medicine, Tokyo 160-8582, Japan

## Abstract

**Background:**

The aim of the present cross-sectional study was to assess possible associations between osteopontin (OPN), and thrombin-cleaved (N-half) OPN, and nephropathy and coronary artery disease (CAD) in patients with type 2 diabetes mellitus (T2DM).

**Methods:**

Plasma levels of OPN, N-half OPN, and high-sensitivity C-reactive protein (hsCRP) were determined in 301 diabetic patients with (n = 226) or without (n = 75) angiographically documented CAD (luminal diameter narrowing >50%), as well as in 75 non-diabetic controls with normal angiography. The estimated glomerular filtration rate (eGFR) was calculated in all patients.

**Results:**

Plasma levels of OPN and hsCRP were significantly higher in patients with T2DM compared with controls. In addition, there was a higher occurrence of moderate renal insufficiency and lower eGFR in patients with T2DM (all *P *< 0.01). T2DM patients in whom OPN levels were greater than the median value had higher serum creatinine levels, a greater prevalence of mild or moderate renal insufficiency, a higher incidence of CAD, and lower eGFR (all *P *< 0.05) than T2DM patients in whom OPN levels were the same as or lower than the median value. However, there were no differences in these parameters when patients were stratified according to plasma N-half OPN levels. Furthermore, there was a significant correlation between OPN, but not N-half OPN, and the severity of nephropathy and CAD in diabetes. After adjustment for potential confounders and treatments, multiple linear regression analysis demonstrated an independent association between OPN, but not N-half OPN, and eGFR. Multivariate logistic regression revealed that higher OPN levels conferred a fourfold greater risk of renal insufficiency and CAD in patients with T2DM.

**Conclusions:**

The results of the present study demonstrate that there is an independent association between plasma levels of OPN, but not N-half OPN, and the presence and severity of nephropathy and CAD in diabetes.

## Background

Micro- and macro-vasculopathies, such as nephropathy and coronary artery disease (CAD), respectively, are common in diabetes and constitute the major causes of death for these patients [1,2]. Proinflammatory cytokines play a critical role in the pathogenesis of diabetic complications through various biochemical and cellular pathways [1,3-5]. Full-length osteopontin (OPN), a macrophage chemotactic protein, was originally identified as a mediator involved in bone remodeling, chronic inflammatory and autoimmune diseases [6,7], and subsequently demonstrated to play an important role in the development of cardiovascular diseases [6,8]. OPN was synthesized by monocytes/macrophages, endothelial cells, and vascular smooth muscle cells, and proved to express in neointima and calcified atheromatous plaque [6,9]. In contrast, an OPN neutralizing antibody could inhibit rat carotid neointimal formation after endothelial denudation [10]. Clinically, a significant association has been demonstrated, independent of traditional risk factors, between plasma OPN levels and atherosclerotic plaque formation in general patients [11,12]. Furthermore, OPN expression has been shown to be upregulated in the vascular wall of diabetic patients and diabetic animal models, which might be induced by high glucose and advanced glycation endproduct [9,13-15]. In kedney, OPN has also been identified in glomerular mesangial cells, podocytes, and endothelial cells [16-18]. Microarray analyses of diabetic versus normal kidneys identified OPN as one of the major genes upregulated in humans with diabetic nephropathy and in mice with either type 1 diabetes or the type 2 db/db model of diabetes [19]. OPN knockout mice were protected from diabetes-induced albuminuria and mesangial expansion [18]. A strong correlation between higher OPN levels and more severe diabetic albuminuria and glomerulosclerosis has been demonstrated in various models of diabetic nephropathy [19,20]. All these observations suggested a possible role of OPN in accelerated atherogenesis and the development of renal disease in diabetes mellitus.

Structurally, OPN contains several cell-interacting domains, as well as protease-cleavage sites, that may be important in regulating its activity. These domains include: (i) an arginine-glycine-aspartate (RGD)-containing domain that interacts with cell surface integrins αvβ3, αvβ1, αvβ5, and α8β1, promoting the migration and/or growth potential of lymphocytes, macrophages, endothelial cells, and vascular smooth muscle cells; and (ii) a cryptic serine-valine-valine-tyrosine-glutamate-leucine-arginine (SVVYGLR)-containing domain cleaved by thrombin that interacts with α9β1, α4β1, and α4β7 integrins and mediates cell adhesion to the NH_2_-terminal fragment of OPN, causing inflammation in an RGD-independent manner [6,21,22]. Patients with diabetes often have a chronic procoagulant state, as reflected by increased thrombin activity and elevated circulating thrombin/anti-thrombin complexes [23-25]. Thrombin-cleaved (N-half) OPN levels are elevated in the vitreous fluid of patients with diabetic retinopathy [26], as well as in the synovial fluid and plasma of patients with rheumatoid arthritis [7,27], highlighting the possibility that this cytokine may be involved in local inflammation. Because diabetes per se is an inflammatory process with increased cytokine levels and enhanced thrombin activity in the vascular wall [1,23], in the present study we tested the hypothesis that there may be an association between both OPN and N-half OPN levels and the presence and severity of nephropathy and CAD in patients with type 2 diabetes mellitus (T2DM).

## Methods

The study protocol was approved by the Ruijin Hospital ethics committee. Written informed consent was obtained from all patients prior to their inclusion in the study.

### Study population

The present study was performed on 301 consecutive diabetic patients (171 men and 130 women) undergoing angiography for suspected CAD or percutaneous coronary intervention (PCI) between June 2005 and October 2007. Patients with concomitant valvular heart disease, cardiomyopathy, acute renal failure, acute and chronic viral or bacterial infections, asthma, tumors, or connective tissue diseases, and those undergoing coronary artery bypass surgery or on dialysis, were excluded from the study. In addition, patients with acute coronary syndrome, in-stent restenosis or those with symptomatic heart failure were excluded from the study. Patients with type 1 diabetes mellitus were identified on the basis of C-peptide measurements and were also excluded from the study. T2DM was diagnosed using the following criteria: (i) fasting plasma glucose levels ≥7.0 mmol/L on two occasions; (ii) two 2-h postprandial plasma glucose readings ≥11.1 mmol/L after a glucose load of 75 g; (iii) two casual glucose readings ≥11.1 mmol/L; or (iv) treatment with oral hypoglycemic drugs or parenteral insulin. The clinical characteristics and risk factors for CAD were recorded for each patient. Almost all T2DM patients were receiving antidiabetic treatment and most were taking statins and angiotensin-converting enzyme (ACE) inhibitors or angiotensin receptor blockers (ARBs).

Seventy-five consecutive non-diabetic patients (38 men, 37 women) who underwent coronary angiography for suspected CAD but had normal coronary arteries served as the control group. The professional activities and diet styles of the control group were matched with those of the diabetic patients. Liver and renal function tests were normal.

To elucidate the relationship between OPN or N-half OPN and nephropathy or CAD in diabetes, T2DM patients were divided into two groups, those with higher than median plasma values of OPN (235.0 ng/mL) and N-half OPN (25.0 pmol/L) and those with median or lower plasma values of OPN and N-half OPN. This was done because the distribution of plasma values of OPN and half-OPN is not normal.

### Coronary angiography

Selective coronary angiography was performed using a femoral or radial artery approach by cardiologists blinded to the study protocol. Significant CAD was diagnosed visually if the narrowing of the luminal diameter of a major epicardial coronary artery was ≥50%. Patients with significant CAD were further classified into those with one-, two-, or three-vessel disease depending on the number of coronary arteries involved. A ≥50% narrowing of the left main coronary artery was considered as two-vessel disease.

### Biochemical investigations

Blood samples were collected from all patients before coronary angiography and after an overnight fast. Plasma OPN level was determined with a commercially available ELISA kit (Human OPN assay kit, IBL, Gunma, Japan) according to its protocol, which only detects full-length OPN. The sensitivity for OPN was 3.33 ng/ml with an intra- and inter-assay CV of <5% and <10%, respectively. Plasma levels of N-half OPN were measured using an ELISA kit (Human Osteopontin N-Half Assay Kit, IBL, Gunma, Japan). In brief, 1:2 diluted testing samples were incubated in the N-half OPN antibody pre-coated wells at 37°C for 1 h. Following washing, 100 uL of labeled OPN antibody solution was added into each well and incubated for 30 min at 4°C. After washing, tetramethyl benzidine was used as a coloring agent, and the absorbance at 450 nm was measured with an automatic ELISA reader (Bio-Rad, Segrate, Italy). The intra- and inter-assay coefficients of variation (CV) were <5% and <8%, respectively, with a sensitivity 3.09 pg/L. Furthermore, plasma levels high-sensitivity C-reactive protein (hsCRP) were determined (Quantikine ELISA kits; R&D Systems, Minneapolis, MN, USA). Estimated glomerular filtration rate (eGFR) was calculated using the formula of the Modification of Diet in Renal Disease study group [26]. Mild, moderate, and severe renal insufficiency was diagnosed when the eGFR was <90, <60, and <30 mL/min per 1.73 m^2^, respectively.

### Statistical analysis

All statistical analyses were performed using SPSS for Windows 13.0 (SPSS Inc., Chicago, IL, USA). Unless indicated otherwise, data are presented as frequencies or percentages for categorical variables and as the mean (±SD) or median (interquartile range) for continuous variables. For categorical clinical variables, differences between groups were evaluated with the Chi-squared test. For continuous variables, normal distribution was evaluated with the Kolmolgorov-Smirnov test. Variables that were not normally distributed were log transformed when necessary. Comparisons between groups were made using analysis of variance for variables with a parametric distribution. Correlations were evaluated by calculating Spearman's correlation coefficient. Multiple linear regression analyses with eGFR and OPN as dependent variables were used to assess independent relationships. A multivariate logistic regression model was constructed to assess the independent determinants for a moderate decrease in eGFR, CAD and either of the two in diabetic patients. Two-sided *P *≤ 0.05 was considered significant.

## Results

### Clinical characteristics

Compared with non-diabetic controls, T2DM patients were older, had higher systolic and diastolic blood pressures, higher triglyceride and fasting glucose levels, and lower high-density lipoprotein-cholesterol levels. More diabetic patients were receiving treatment with statins and ACE inhibitors or ARBs than controls (Table 1).

**Table 1:** Clinical characteristics and biochemical assessments of study patients

Variable	Control (n = 75)	Diabetes (n = 301)
Men/Female (n)	38/37	171/130
Age (years)	56.7 ± 10.5	65.5 ± 9.7*
Smokers (%)	14 (18.7)	88 (29.2)
Hypertension (%)	6 (8.0)	252 (83.7)*
SBP (mmHg)	122 ± 15	136 ± 19*
DBP (mmHg)	75 ± 8	79 ± 11^†^
Hyperlipidemia (%)	25 (33.3)	196 (65.1)*
Triglyceride (mmol/L)	1.35 ± 0.65	2.08 ± 1.63*
Total cholestrol(mmol/L)	4.30 ± 0.80	4.47 ± 1.00
HDL-C (mmol/L)	1.33 ± 0.35	1.17 ± 0.31*
LDL-C (mmol/L)	2.49 ± 0.55	2.55 ± 0.82
Lipoprotein (a) (g/L)	0.23 ± 0.19	0.25 ± 0.20
Fast glucose (mmol/L)	4.85 ± 0.63	6.98 ± 2.56*
HbA1c (%)	-	7.57 ± 1.35
ACEI or ARB (%)	7 (9.3)	234 (77.7)*
Statins (%)	17 (22.7)	237 (78.7)*

Data are given as either the number of patients (%), mean ± SD. *P < 0.001, ^†^*P *< 0.01 compared with the control group.

Abbreviations: SBP, systolic blood pressure; DBP, diastolic blood pressure; HDL-C, high-density lipoprotein-cholesterol; LDL-C, low-density lipoprotein-cholesterol; HbA1c, glycated hemoglobin; ACEI, angiotensin-converting enzyme inhibitor; ARB, angiotensin II receptor blocker

### OPN and N-half OPN levels in relation to nephropathy and CAD

Compared with controls, diabetic patients had significantly higher OPN (*P *< 0.001), hsCRP (*P *< 0.001), and creatinine (*P *< 0.01) levels, were more likely to have moderate renal insufficiency (*P *< 0.01), and had a lower eGFR (*P *< 0.001; Table 2).

**Table 2:** Osteopontin (OPN) and thrombin-cleaved osteopontin (N-half OPN) in relation to nephropathy and coronary artery disease

Variable	Control (n = 75)	Diabetes (n = 301)	OPN ≤ median (n = 150)	OPN >median (n = 151)	N-half OPN ≤ median (n = 150)	N-half OPN >median (n = 151)
Renal function parameters						
Creatinine (μmol/L)	74.7 ± 14.6	84.8 ± 26.1^†^	79.2 ± 19.5	90.3 ± 30.2*	83.9 ± 27.3	85.8 ± 24.8
eGFR (mL/min/1.73 m^2^)	89.6 ± 20.3	79.8 ± 20.9*	84.1 ± 19.5	75.5 ± 21.3*	80.9 ± 20.2	78.6 ± 21.5
eGFR <90 mL/min/1.73 m^2 ^(%)	45 (60)	206 (71.3)	91 (63.6)	115 (78.8)^†^	106 (72.6)	100 (69.9)
eGFR <60 mL/min/1.73 m^2 ^(%)	2 (2.7)	48 (16.6)^†^	16 (11.2)	32 (21.9)^‡^	19 (13.0)	29 (20.3)
CAD (%)	-	226 (75.1)	104 (69.3)	122 (80.8)^‡^	111 (74.0)	115 (76.2)
Inflammatory cytokines						
OPN (ng/mL)	218 (190, 261)	235 (201, 284)*	201 (186, 218)	284 (258, 359)*	242 (212, 279)	226 (191, 286)
N-half OPN (pmol/L)	23.8 (18.9, 41.9)	25.3 (20.6, 34.9)	27.9 (20.8, 42.7)	23.8 (20.4, 31.4)^†^	20.6 (18.7, 22.3)	34.8 (29.3, 44.3)*
HsCRP (ng/mL)	150 (102, 330)	286 (139, 675)*	206 (130, 373)	426 (173,1049)*	285 (149, 629)	288 (130, 790)

Data are given as mean ± SD or the median (interquartile ranges), as appropriate. *P < 0.001, ^†^*P *< 0.01, ^‡^*P *< 0.05 compared with the control group, the group with OPN values below the median value and the group with N-half OPN values below the median, respectively.

Abbreviations: eGFR, estimated glomerular filtration rate; CAD, coronary artery disease; OPN, osteopontin; N-half OPN, Thrombin-cleaved N-half osteopontin; hsCRP, high sensitive C-reactive protein.

**Figure 1:**
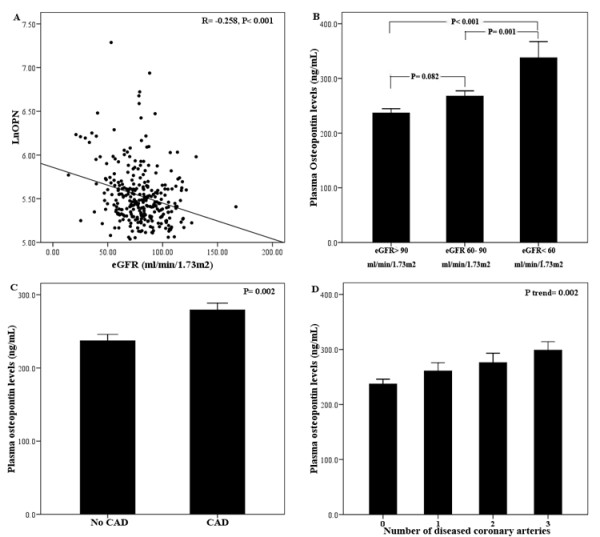
**Association between plasma osteopontin (OPN) levels and renal insufficiency and coronary artery (CAD) disease in all diabetic patients**. (A) Scatter plot showing the relationship between OPN and estimated glomerular filtration rate (eGFR) in the diabetic population. OPN concentrations were log-transformed and *R *represents Pearson's correlation coefficient. (B) Plasma OPN levels in patients with different renal function stratified according to eGFR. Data are the mean ± SEM. Differences among groups were analyzed by one-way ANOVA followed by post hoc analysis (with Bonferroni's correction). (C, D) Relationship between plasma OPN levels and the presence (C) and severity (D) of CAD in diabetes. Data are the mean ± SEM.

Serum creatinine and occurrence rate of mild or moderate renal insufficiency were higher in T2DM patients with OPN levels greater than the median compared with those with OPN levels lower than the median (creatinine 90.3 ± 30.2 vs. 79.2 ± 19.5 μmol/L, respectively (*P *< 0.001); mild renal insufficiency 78.8% vs. 63.6%, respectively (*P *< 0.01); and moderate renal insufficiency 21.9% vs 11.2%, respectively (*P *< 0.05)); in contrast, eGFR was higher in T2DM patients with OPN levels below the median (84.1 ± 19.5 vs 75.5 ± 21.3 mL/min per 1.73 m^2^; *P *< 0.001; Table 2). There were no significant differences in creatinine levels, eGFR, and the occurrence of renal insufficiency between groups stratified according to N-half OPN values (Table 2). In addition, there was a negative correlation found between OPN, but not N-half OPN, and eGFR in T2DM patients (*r *= -0.258; *P *< 0.001; Figure 1A), as well as stepwise increases in mean OPN concentrations in patients with deteriorating renal function (237 ± 72, 268 ± 120, and 338 ± 203 ng/mL in T2DM patients with normal, mild, moderate renal insufficiency, respectively; *P *< 0.001; Figure 1B). In the present study, the five T2DM patients whose eGFR was <30 mL/min per 1.73 m^2 ^had highest plasma OPN levels (402 ± 141 ng/mL). However, there was no difference in the percent of N-half OPN (N-half OPN/OPN*100%) in T2DM patients with normal, mild and moderate renal insufficiency (*P *= 0.65).

Patients with OPN concentrations greater than the median value had a higher incidence of CAD than those with OPN levels below the medium value (80.8% vs. 69.3%, respectively; *P *< 0.05), but differences were not found for patient groups stratified according to N-half OPN values (76.2% vs. 74.0%; *P *> 0.05; Table 2). Furthermore, in patients with T2DM, the presence and severity of CAD were significantly associated with plasma OPN (*P *= 0.002 and *P*_trend _= 0.002), but not with N-half OPN levels (Figure 1C, D). In addition, the percent of N-half OPN was similar in T2DM patients with and without CAD (*P = 0.55*).

In T2DM patients, there was a positive correlation between plasma OPN levels and hsCRP (*r *= 0.394; *P *< 0.001). After adjusting for age, the associations between plasma OPN and hsCRP (*r *= 0.383; *P *< 0.001) remained significant. However, N-half OPN did not correlate with other variables in this study.

### Multivariate regression analysis on nephropathy and CAD in diabetes

Multiple linear regression analysis showed that after adjustment for several potential confounding variables in Model A, eGFR was independently correlated with OPN (*P *= 0.002). In a similar analysis, when OPN was used as a dependent variable in Model B after adjustment for several potential confounding variables, independent associations were found between OPN and N-half OPN, hsCRP, and eGFR (all *P *< 0.01; Table 3).

**Table 3:** Multiple linear regression analysis of relationships between selected variables and estimated glomerular filtration rate or osteopontin

Variable	β	SEM	*P*
Model A: Dependent variable eGFR			
Age	-0.889	0.127	< 0.001
OPN	-11.967	3.823	0.002
Model B: Dependent variable OPN			
N-half OPN	-0.136	0.027	< 0.001
hsCRP	0.103	0.017	< 0.001
eGFR	-0.003	0.001	0.002

In addition to the significant covariables shown in the table, Model A also included sex, cigarette smoking, systolic blood pressure (SBP), total cholesterol, triglyceride, lipoprotein (a), fasting glucose, use of angiotensin receptor blockers (ARBs) or angiotensin-converting enzyme inhibitors (ACEI), use of statins, log-transformed thrombin-cleaved osteopontin (N-half OPN), and log-transformed high-sensitivity C-reactive protein (hsCRP). Model B included sex, age, cigarette smoking, SBP, total cholesterol, triglyceride, lipoprotein (a), fasting glucose, use of ARBs or ACEI, and the use of statins.

Multiple logistic regression analysis revealed that after adjustment for several potential confounding variables, the risk of a moderate reduction in eGFR, the presence of CAD, and a moderate reduction in eGFR or CAD increased more than fourfold (*P *= 0.006, *P *= 0.034, and *P *= 0.027, respectively) for OPN in the diabetic cohort (Table 4). Furthermore, eGFR was independently associated with the presence of CAD (*P *= 0.026; Table 4).

**Table 4:** Determinants for presence of an estimated glomerular filtration rate <60 mL/min per 1.73 m^2 ^and coronary artery disease

Variable	Odds ratio	95% Confidence interval	*P*
Model A: Dependent variable moderate reduction in eGFR			
Age	1.08	1.04-1.14	0.001
OPN	4.58	1.55-13.59	0.006
Model B: Dependent variable CAD			
Sex	2.29	1.10-4.79	0.028
Statins	5.14	2.52-10.48	< 0.001
OPN	4.30	1.12-16.54	0.034
eGFR	0.20	0.05-0.82	0.026
Model C: Dependent variable moderate reduction in eGFR or CAD			
Age	1.06	1.02 - 1.11	0.002
Statins	4.62	2.29 - 9.35	<0.001
OPN	4.49	1.18 - 17.05	0.027

Models A and C also included sex, cigarette smoking, systolic blood pressure (SBP), total cholesterol, triglyceride, lipoprotein (a), fasting glucose, use of angiotensin receptor blockers (ARBs) or angiotensin-converting enzyme inhibitors (ACEI), use of statins, log-transformed thrombin-cleaved osteopontin (N-half OPN), and log-transformed high-sensitivity C-reactive protein (hsCRP). Model B also included cigarette smoking, SBP, total cholesterol, triglyceride, lipoprotein (a), fasting glucose, use of ARBs or ACEI, log-transformed N-half OPN, log-transformed hsCRP, and log-transformed estimated glomerular filtration rate (eGFR).

## Discussion

The present study demonstrated that plasma levels of OPN, but not N-half OPN, are independently associated with the presence and severity of nephropathy and CAD in diabetes.

### Relationship between plasma OPN levels and nephropathy and CAD in diabetes

Previous studies have shown that advanced glycation end-products and angiotensin II can stimulate OPN synthesis by a variety of cells, including mesangial cells and podocytes, and initiate local effects of cell spreading, adhesion, and proliferation [28-30]. In rat models of streptozotocin- or high-fat diet-induced diabetic nephropathy, OPN expression is significantly upregulated in the renal cortex and aorta [31,32], and increased OPN expression is strongly correlated with severe diabetic albuminuria and glomerulosclerosis [19,20]. In contrast, OPN-knockout mice do not develop albuminuria in response to lipopolysaccharide, and are protected from diabetes-induced mesangial expansion [18]. In patients with diabetic nephropathy, OPN expression is closely related to the degree of cortical interstitial scarring [33]. Yamaguchi et al. found that plasma OPN levels were increased in diabetic patients with renal failure [34]. In the present study, all patients had cardiovascular risk factors and had been hospitalized for diagnostic angiography and/or PCI. We found that plasma OPN concentrations were proportional to the severity of renal dysfunction and were an independent risk factor for the presence and severity of renal insufficiency, suggesting that OPN may play a pivotal role in the development of diabetic nephropathy in these high-risk patients. Conversely, the presence of renal insufficiency could lead to elevated plasma OPN concentrations, forming a vicious cycle that exaggerates diabetic nephropathy and atherosclerosis.

Plasma OPN levels have been shown to be correlated with the angiographic severity of CAD, independent of conventional risk factors [12], and have been identified as a potential marker of atherosclerotic disease progression and adverse cardiovascular outcome in patients with stable angina [11]. Consistent with previous observations [35], we found in the present study that OPN was closely associated with hsCRP levels independent of age, suggesting that OPN, as an impaortant inflammatory cytokine, may aggravate the inflammation status in diabetes mellitus. In addition, we demonstrated a significant association between plasma OPN levels and the presence and severity of CAD in diabetic patients, indicating that OPN may be critically involved in the inflammatory processes that take place within the vascular wall in diabetes.

### Relationship between plasma concentrations of N-half OPN and nephropathy and CAD in diabetes

In vitro studies have reported that the N-terminal fragments of OPN generated by both thrombin cleavage and matrix metalloproteinase (MMP) cleavage induce enhanced adhesion compared with the effects of full-length OPN. This appears to be due mostly to increased activity of the RGD site, perhaps an indication of conformational change resulting in higher affinity binding [6]. In a model of rheumatoid arthritis, an antibody specifically neutralizing only the SLAYGLR domain of mouse OPN (homologous to the SVVYGLR of human OPN) greatly abrogated monocyte migration towards the thrombin-cleaved form of OPN and inhibited the proliferation of synovial cells, bone erosion, and inflammatory cell infiltration in arthritic joints [21]. Another study showed that the SVVYGLR sequence induces pro-MMP9 expression in isolated vascular smooth muscle cells, as well as in aortas from diabetic mice [36]. Clinically, N-half OPN levels are elevated in the synovial fluid, urine, and plasma of patients with rheumatoid arthritis [7,27,35], as well as in the vitreous fluid of those with diabetic retinopathy [26], indicating that this cytokine is involved in local inflammation. Because diabetes per se is an inflammatory process with increased cytokine levels and enhanced thrombin activity in the vascular wall [23], we hypothesized that N-half OPN may be associated with the development and severity of nephropathy and CAD in this setting. Unexpectedly, there was no association between N-half OPN and CAD or hsCRP in diabetic patients. This may be due to the relatively lower concentrations of N-half OPN compared with full-length OPN in plasma (the percent of N-half OPN/OPN was no more than 1%). Alternatively, N-half OPN may exist predominantly in local tissue rather than in the circulation. Further studies are warranted to examine the role of circulating and local vascular N-half OPN in the pathogenesis of diabetic nephropathy and atherosclerosis.

### Clinical relevance of OPN in diabetes

Type 2 diabetes is an independent risk factor for cardiovascular diseases and a CAD equivalent [2,37]. Its complications mainly include diabetic nephropathy, atherosclerosis and retinopathy [1]. Although hypoglycemic agents can control blood glucose, current treatment is still not sufficient to prevent the development of diabetic complications [37]. Therefore, in these high-risk patients, early detection and effective prevention of the complications become an important issue. In the present study, we found that plasma OPN level, but not N-half OPN parallels with the severity of nephropathy and CAD in diabetes, suggesting that an increased plasma OPN level may be used as an indicator for screening diabetic vasculopathy. Furthermore, since basic research showed that OPN contributes to the development of nephropathy and atherosclerosis in diabetes [10,18-20], inhibition of OPN may become a novel therapeutic strategy for these paitents.

### Study limitations

There are several limitations to the present study. First, the present study was a cross-sectional design; thus, our results reflect only the association between OPN or N-half OPN levels and prevalent rather than incident atherosclerosis. Second, all subjects in the present study were scheduled for coronary angiography and/or PCI, and are thus likely to be more typical of a high-risk patient group than the healthy population at large.

In conclusion, the present study indicates that plasma concentrations of OPN, but not N-half OPN, are independently associated with the presence and severity of nephropathy and CAD in T2DM patients. Approaches involving the inhibition of OPN signaling may prove valuable therapeutic strategies for the prevention of diabetic complications and improved patient outcomes.

## Competing interests

The authors declare that they have no competing interests.

## Authors' contributions

XY contributed to the study design, collected the data, contributed to the discussion, and wrote the manuscript. LL, WW, LW, QZ, RZ, and QC collected the data. MS, KF reviewed/edited the manuscript. WS contributed to the study design, discussion, and reviewed/edited the manuscript. All authors have read and approved the final manuscript.
